# Insects and their Laboulbeniales (Ascomycota, Fungi) of Lake Eustis and Emeralda Marsh Conservation Area: A case study on urbanization and diversity

**DOI:** 10.1002/ece3.8246

**Published:** 2021-11-19

**Authors:** Patricia J. Kaishian

**Affiliations:** ^1^ Department of Environmental and Forest Biology SUNY College of Environmental Science & Forestry Syracuse New York USA; ^2^ Department of Botany and Plant Pathology Purdue University West Lafayette Indiana USA

**Keywords:** anthropogenic disturbance, biodiversity estimator, climate change, ecology, fungal interaction, insect, mycology, urbanization

## Abstract

A rapid biodiversity assessment of insects and associated Laboulbeniales fungi was conducted over the course of five nights in August, 2018, at two central Florida lakes: Lake Eustis and the nearby protected and restored National Natural Landmark, Emeralda Marsh Conservation Area (EMCA), which encompasses a portion of Lake Griffin. Lake Eustis was surveyed for Laboulbeniales in 1897 by mycologist Dr. Roland Thaxter but has not since been investigated. Because Lake Eustis has been urbanized, with the lake perimeter almost entirely altered by human development, the site offers a look into Laboulbeniales diversity across a 121‐year timeline, before and after human development. By surveying Lake Eustis and EMCA, a modern case study comparison of Laboulbeniales and insect diversity between a developed and a protected and restored system is made. A total of 4022 insects were collected during the rapid assessment. Overall, insect abundance was greater at EMCA, with 3001 insects collected, than 1021 insects collected from Eustis. Although family‐level insect richness was comparable between sites, with 55 families present at EMCA and 56 at Eustis, 529 out of 3001 (17.6%) of the insects collected at EMCA were hosts to parasitic Laboulbeniales fungi, whereas only 2 out of 1021 (0.19%) collected from Eustis were infected. A total of 16 species of Laboulbeniales found at EMCA compared with only one at Eustis. The current number of Laboulbeniales species documented at Eustis was incredibly depauperate compared with the 26 species and two varieties recorded by Thaxter in 1897. These findings suggest the possibility of utilizing Laboulbeniales as indicators of ecosystem health, and future research should investigate this question further. A figure displaying host–parasite records and a species list of Laboulbeniales are presented. Finally, updated occurrence records for species of *Ceratomyces* and *Hydrophilomyces* are provided.

## Graphical abstract

Two lakes in central Florida were surveyed for insects and the associated fungal parasites, Laboulbeniales. One of the lakes had been the site of intensive restoration efforts while the other lake is highly developed and un‐restored. This study found stark differences in fungal diversity between the sites, and explores the possibility of the use of Laboulbeniales fungi as indicators of environmental health.

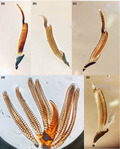

## 1 INTRODUCTION

### 1.1 Insect diversity

Assessing biodiversity of insects and fungi presents challenges as they are two most diverse groups of eukaryotes and also suffering a paucity of trained taxonomists. Knowledge of insects and fungi can be described as highly uneven, with representative members, often those associated with agriculture, industry, or disease, receiving vastly more attention than other groups (Ainsworth et al., 2018; Kim, 1993). In both groups, millions of species remain undescribed (Grimaldi & Engel, 2004; Hawksworth & Lücking, 2017).

Insect diversity studies have yielded a range of estimates for global and site‐specific studies, with a number of researchers trying their hand at different techniques and methods in order to arrive at sound estimates. Insects are so diverse that researchers do not even agree on estimates of currently described species. Estimates range from 750,000 (Wilson, 1992) to 1.4 million (Hammond, 1992). Based on work by Gaston (1991) and Resh and Cardé (2003), Grimaldi and Engel (2004) endorse the estimate of 925,000 for currently named species. Although this discrepancy may seem surprising, it is also understandable, given the lack of sufficient incentives for researchers to spend time scouring old literature, synonymizing, cataloging, and producing monographs.

Regarding estimates of living species, both described and undescribed, estimates of insect richness are even more variable. The lowest estimate is about 2 million species (Grimaldi & Engel, 2004), and the largest is a staggering 30 million tropical insect species (Erwin, 1982). Erwin's estimate was based onfogging techniques on tree canopies in neotropical forests, upon which extrapolations were made for total insect diversity. Erwin recorded trees as having unique species of insects in their canopies and used the total tropical tree diversity of approximately 50,000 species to extrapolate. Most researchers now agree this estimate is much too high largely because the assumption that the insects found in tree canopies would be highly host‐specific is likely erroneous (Grimaldi & Engel, 2004). Grimaldi and Engel endorse Gaston’s (1991) estimate of about 5 million total living insect species. This estimate was based on surveying collections held by systematists around the world. Despite this method having some potential shortcomings, for example, collection biases of individual collectors and the presence of unexamined or unknown duplicates held across collections, the authors believe it is currently the most accurate estimate of global insect diversity. If this estimation is accepted, then the aforementioned figure of 925,000 named insects would represent 20% of extant insect diversity.

In a quickly changing climate, there is increasing evidence of mass declines of insects and, therefore, a pressing need to monitor insect biodiversity at local and regional scales (Kim & Byrne, 2006). Comparable data have not been recovered for fungi as fungal conservation is in its early stages (Mueller, 2017). However, there is indication of declines in fungal species richness in response to human disturbances, including but not limited to nutrient loading, mass tree die‐off due to introduced pathogens, acidification, and habitat loss (Arnolds, 1991; Treu et al., 2014). Biodiversity studies usually focus on vertebrate animals and vascular plants, whereas those focused on invertebrates and fungi are rare (Fiesler & Drake, 2016). Despite being ubiquitous and essential components of the biosphere, macroinvertebrates such as insects remain underserved with respect to their risk assessment and conservation status. As of 2006, <0.1% of described insects had been assessed for inclusion in the Red List maintained by the International Union for the Conservation of Nature (IUCN) (Rodrigues et al., 2006).

Because of the staggering diversity and abundance of insects, there exists feasibility concerns when designing biodiversity studies. Rapid biodiversity assessments of insects are frequently employed in order to glean broad but manageable data sets that can be, albeit tentatively, extrapolated as a flexible measure of communities and populations (Ward and Larivière, 2004). There is currently a concerted effort by entomologists to establish optimal sampling methods for assessing insect biodiversity by taxa, population, assemblage, community, habitat, and region (Brown, 1997; Hughes et al., 2000; Kim, 1993; Ward and Larivière, 2004). Many scientists agree that establishing protected areas is the most effective way to protect multikingdom species diversity, particularly when considering understudied, vulnerable, and uncharismatic groups, which includes many insects and fungi (Hughes et al., 2000).

### 1.2 Fungal diversity

The state of knowledge of fungi is substantially behind that of insects. A widely cited estimate of global fungal diversity is upward of 1.5 million (Hawksworth, 1991). Mycologists generally agree this is a conservative estimate, in part because it was based primarily on extrapolations from fungus‐to‐plant ratios in temperate regions and did not give due consideration to the hyperdiverse realm of insect‐associated fungi, such as Laboulbeniales, or account for tropical species diversity (Hawksworth, 1991; Hawksworth & Lücking, 2017). The highest estimate of fungal diversity is currently 6 million, which was put forward by Taylor et al. (2014). The most recent estimate (Hawksworth & Lücking, 2017) of extant fungi is 2.2 to 3.8 million, and the updated fungus‐to‐plant ratio for temperate zones is 8:1. Of that, ~138,000 species have been described (Hibbett et al., 2016; Kirk, 2019). With only ~6% of the lower estimation being known to science, the remaining task is tremendous. Unlike many plant and animal groups, fungi do not broadly enjoy the benefits of being well‐studied and clearly understood. New species are most likely to be discovered by investigating relatively understudied habitats and microhabitats, including insect bodies, lichen‐dwelling fungi, and cryptic species, through environmental (eDNA) sequencing (Hawksworth & Lücking, 2017) and within natural history collections (Wijayawardene et al., 2020).

In addition to fungi being relatively poorly studied, the often sporadic, ephemeral, and unpredictable appearance of fruiting bodies complicates obtaining baseline data on factors such as occurrence and abundance and has constrained our ability to provide clear objective assessments of fungi overtime. The complex biotic and abiotic forces leading to a species even producing a fruiting body remains unknown in many cases and likely involves the combination and interactions of degree days, soil temperature, precipitation volume, vegetation patterns, and so forth (Mihail et al., 2007). Although some fungi, for example, some species of morels, can be reliably found in the same place at more or less the same time every year, other species, such as *Ionomidotis* sp. (personal observation) or *Hericium bembedjaense* (Jumbam et al., 2019) may be seen once in a given location and then not again for years, if ever. Although substantial efforts have recently been made in fungal conservation, this field remains in its early stages (Mueller, 2017). According to the State of the World's Fungi (Ainsworth et al., 2018), only 56 species of fungi have been evaluated for placement on the IUCN Red List, of which 43 ended up being included. Comparatively, 25,452 species of plants and 68,054 species of animals have been evaluated. It is therefore imperative that fungi receive increased attention, concern, and action.

### 1.3 Laboulbeniales

Laboulbeniales (Ascomycota, Fungi) are microscopic obligate parasites on arthropods, primarily occurring on insects. Laboulbeniales are considered the most diverse lineage of insect‐associated fungi, with ~2325 described species in 145 genera, but current estimates indicate there are at least 40,000 species awaiting description (Haelewaters et al., 2020; Kirk, 2019; Weir & Hammond, 1997). The impact of Laboulbeniales fungi on their insect hosts are not fully understood, and basic studies of their biology are still limited (Haelewaters et al., 2021). Only a handful of scientists in the world specialize in the study of Laboulbeniales, and yet because of the size, diversity, and uniqueness of this lineage, it is undoubtedly a cradle of novel taxonomic and ecological information.

One early and prolific researcher of Laboulbeniales was Dr. Roland Thaxter. During his career at Harvard University between 1891 and 1932, Thaxter described >1000 species of Laboulbeniales and made substantial contributions to our understanding of their development and general biology. Most of his life's work on Laboulbeniales is contained within a five‐volume set of his *Contribution towards a Monograph of the Laboulbeniaceae* (1896, 1908, 1924, 1926, 1931), a tremendous contribution to mycology.

### 1.4 Foundation of study

One of Thaxter's collection sites for Laboulbeniales was in Eustis, a small central Florida city on the east shore of Lake Eustis. Beginning in the early 1800s, colonial Europeans forcefully established Eustis on Seminole land (Preserving Eustis History, n.d.). The city was named after General Abraham Eustis, who was known for his role in wars against the Seminole people (Preserving Eustis History, n.d.). The numerous connected waterways in the region allowed for Eustis to become a hub for steamboats, and the construction of the railroad that connected many Floridian towns in 1880 led to an increase in settlement from <500 in 1900 to 21,300 in present day Eustis.

According to his travel records, Thaxter was in Eustis during the very early days of the city, from September 25th to October 10th, 1897 (Pfister, 1982), before most of the present urbanization. Because the methods employed by Thaxter during this trip are unpublished and unrecorded, it is unclear precisely how much time was spent collecting or the precise locations from which he collected (D. H. Pfister, personal communication). Throughout Thaxter's work, 26 species and two varieties of Laboulbeniales were recorded and/or described from Eustis, Florida (Table 1): *Autoicomyces acuminatus*, *Cantharomyces pusillus*, *Ceratomyces ansatus*, *C. camptosporus*, *C. cladophorus*, *C. confusus*, *C. filiformis*, *C. floridanus*, *C. longicornis*, *C. minisculus*, *C. mirabilis*, *Chitonomyces affinis*, *Ch. dentiferus*, *Ch. distortus*, *Ch. floridanus*, *Ch. hydropori*, *Ch. lichanophorus*, *Ch. occultus*, *Ch. paradoxus*, *Ch. psittacopsis*, *Ch. uncigerus*, *H. halipli*, *Hydrophilomyces reflexus*, *Hy. rhynchophorus*, *Laboulbenia retusa*, *L. texana var. rostellata*, *L. texana var. tibialis*, *Rhynchophoromyces elephantinus*, *Teratomyces mirificus*, and *Zodiomyces vorticellarius*.

**TABLE 1:** Species of Laboulbeniales recorded from Eustis in 1897 and 2018 and from EMCA in 2018

Fungus	Host classification	Eustis 1897	Eustis 2018	EMCA 2018
*Autoicomyces acuminatus*	*Berosus* sp.	X		
*Ceratomyces ansatus*	*Tropisternus striolatus*	X		X
*Ceratomyces camptosporus*	*Tropisternus lateralis*	X		
*Ceratomyces cladophorus*	*Tropisternus nimbatus*	X		
*Ceratomyces confusus*	*Tropisternus* sp.	X		X
*Ceratomyces filiformis*	*Tropisternus striolatus*	X		X
*Ceratomyces floridanus*	*Tropisternus glaber*	X		
*Ceratomyces longicornis*	*Tropisternus glaber*	X		X
*Ceratomyces minisculus*	*Tropisternus striolatu*, *T. lateralis*	X		
*Ceratomyces mirabilus*	*Tropisternus glaber*	X		X
*Chitonomyces affinis*	*Laccophilus proximus*	X		
*Chitonomyces dentiferus*	*Laccophilus proximus*	X		
*Chitonomyces distortus*	*Laccophilus maculosus*	X		
*Chitonomyces floridanus*	*Cnemidotus punctatus*	X		
*Chitonomyces hydropori*	*Hydroporus modestus*	X		
*Chitonomyces lichanophorus*	*Laccophilus maculosus*	X		
*Chitonomyces occultus*	*Cnemidotus* sp.	X		
*Chitonomyces psittacopsis*	*Laccophilus proximus*	X		
*Chitonomyces uncigerus*	*Laccophilus* sp.	X		
*Chitonomyces* sp.	*Tropisternus* sp.			X
*Hesperomyces virescens*	Coccinellidae		X	X
*Hydraeomyces halipli*	*Haliplus* sp., *Cnemidotus* sp.	X		
*Hydrophilomyces gracilis*	*Phaenonotum* sp.			X
*Hydrophilomyces hamatus*	*Cercyon* sp.			X
*Hydrophilomyces reflexus*	*Phaenonotum estriatum*	X		
*Hydrophilomyces rhynchophorus*	*Phaenonotum estriatum*	X		
*Laboulbenia retusa*	*Brachinus* sp.	X		
*Laboulbenia texana* var. *rostellata*	*Brachinus* sp.	X		
*Laboulbenia texana* var. *tibialis*	*Brachinus* sp.	X		
*Laboulbenia* sp. 1	Staphylinidae			X
*Laboulbenia* sp. 2	Staphylinidae			X
*Rhynchophoromyces elephantinus*	Undet.	X		
*Teratomyces mirificus*	*Acylophorus flavipes*	X		
*Zodiomyces vorticellarius*	Hydrophilidae (*Anisodactylus* sp., *Patrobus* spp., *Pterostichus* spp. *Platynus* spp.)	X		X

Eustis has changed considerably over the last 100 years, with a majority of the lake perimeter being cleared for housing and other human infrastructure (Google Earth, n.d.). In addition, since Thaxter's visit in 1897, no research has been published dedicated to Laboulbeniales in Eustis, or even from the state of Florida.

**FIGURE 1:**
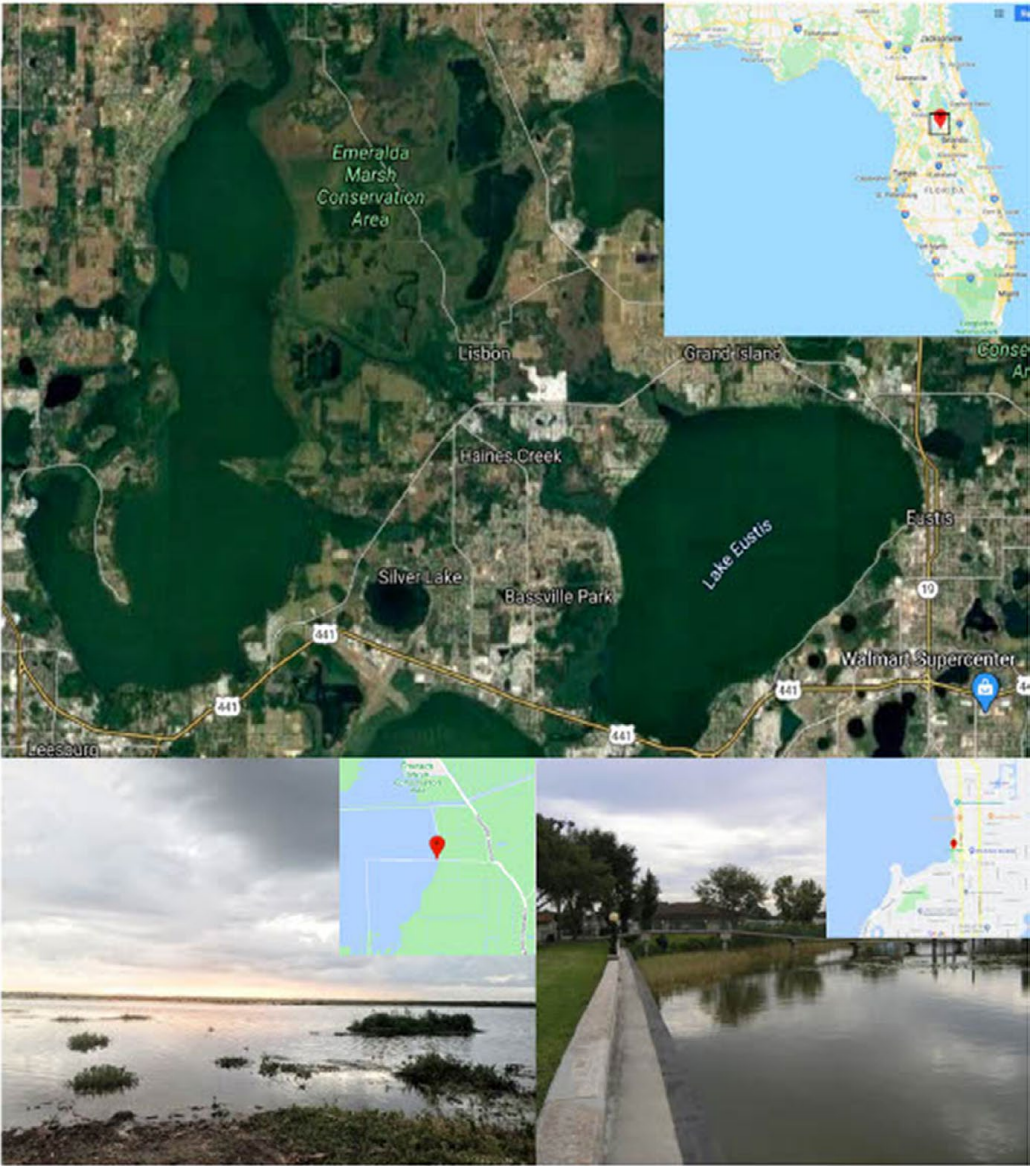
Top: Map of Lake Eustis and Lake Griffin and surrounding area of of Lake County, FL; Bottom Left: Collection site at EMCA with natural littoral zone (photo by author); Bottom Right: Collection site at Lake Eustis showing altered littoral zone (photo source: www.pinterest.com/pin/626422629412672623/)

The overall goal of this study was to conduct a biodiversity assessment over time, as well as between two habitats. By returning to Eustis and attempting to re‐collect species recorded by Thaxter, the goal was to provide insights into shifts in biodiversity of Laboulbeniales and their associated insects since 1897. Because Eustis is now impacted by urbanization, Emeralda Marsh Conservation Area (EMCA), was also sampled as a control. EMCA includes a portion of Lake Griffin and surrounding habitat and is located ~14 km from the east shore of Lake Eustis. Due to its designation as a National Natural Landmark since 1974 and subsequent and ongoing restoration efforts, EMCA provides a closer approximation of the habitat in which Thaxter sampled (Figure 1). Two biodiversity assessments were made: one over time (between Eustis in 1897 and Eustis in 2018) and one between habitats (between Eustis and EMCA in 2018). The working hypothesis for this study was that EMCA, the restored site, would contain greater insect and fungal diversity than Eustis, the unprotected and unrestored site. I further hypothesized EMCA would be more likely to harbor species recorded by Thaxter than Eustis. These data provide a baseline for understanding how urban development around lake systems may affect biodiversity of insects and their accompanying Laboulbeniales parasites, and to begin to explore if and how Laboulbeniales may serve as a proxy for biodiversity and an indicator for ecosystem health.

## 2 METHODS

### 2.1 Site description

Lake Eustis and EMCA (including Lake Griffin) are part of the Central Valley Region (Region 7508) of Florida (Figure 1). Lakes in this subtropical region are categorized by being large, shallow, and eutrophic (Lake County Water Atlas, Emeralda Marsh, n.d.). Lake Eustis and Lake Griffin are part of the Ocklawaha Chain of Lakes, which includes a total of 10 connected lakes. The headwaters of this chain is Lake Apopka, which is fed by a natural spring and by rain. Lake Griffin is the most downstream of the 10 lakes and Lake Eustis is directly upstream from Lake Griffin. Lake Griffin empties northward into the Ocklawaha River, which ultimately connects to the St. Johns River (St. Johns River Water Management District, Lake Apopka Basin, n.d.)

The surface area of Lake Griffin is ~38 km^2^, and that of Lake Eustis is~31 km^2^. The average depth of Lake Griffin is ~2 m, and that of Lake Eustis is ~3 m. The bottom of the Lake Griffin is composed of soft organic matter measuring an average of 1.7 m thickness (Fulton et al., 2015). Equivalent measurements for Lake Eustis were not available. Over the past ~150 years, the Ocklawaha Chain of Lakes have experienced a barrage of human manipulations including, draining, dredging, levying, agricultural conversion, waste dumping, and nutrient loading. In the 1950s, the eastern area of Lake Griffin was levied and drained and converted into agricultural land and muck farms. These farms became an external source of nutrient loading into Lake Griffin (Fulton et al., 2015).

As of 2004, pervious and impervious percentages of the Lake Griffin Basin was 65% and 35%, respectively, whereas the basin containing Lake Eustis (Burrell Basin) was 50% pervious/impervious (Fulton et al., 2004). Surface area coverage by emergent and floating‐leaved vegetation decreased from ~50% in the 1940s to <2% in the 1970s (Fulton et al., 2015). Similar data and exact mapping of wetland and vegetation loss are not available for Lake Eustis; however, mention is made in an issue of Engineering News and American Railway Journal (1884) of Apopka Drainage Company draining ~0.4 km^2^ between Lake Eustis and Lake Dora for farming. Additionally, >405 km^2^ of wetlands and forested uplands were destroyed by human development in Lake County as a whole (Lake County's Comprehensive Plan EAR – Conservation Element, 1997).

Emeralda Marsh was designated as a National Natural Landmark in 1974 (St. Johns River Water Management District, Emeralda Marsh Conservation Area, n.d.). Since at least the early 1980s, Lake Griffin has been hypereutrophic (Fulton, 2015). In the early 1990s, ~12 km^2^ of former muck farms in the EMCA were purchased by the St. Johns River Water Management District (SJRWMD). Over the past 30 years, aquatic and wetland restoration efforts in and around EMCA have focused on revegetation, re‐establishing connectivity with Lake Griffin, and reducing phosphorus and pesticide loading (St. Johns River Water Management District, Emeralda Marsh Conservation Area, n.d.; Fulton et al., 2015). Management and restoration projects are ongoing. Most recently, in 2017 the remaining levees were breached, reconnecting the area to Lake Griffin (St. Johns River Water Management District, Emeralda Marsh Conservation Area, n.d.).

Restoration activities have been extensive at Lake Griffin but comparable efforts have not been made at Lake Eustis (Fulton et al., 2015). Likely as a result of these activities, total phosphorus (TP), chlorophyll‐a, and total nitrogen (TN) have been decreasing with statistical significance in Lake Griffin between 1994 and 2012. Comparatively, Lake Eustis (as well as other lakes in the chain) have not seen significant changes in TP, but did have significant decreases in chlorophyll‐a and TN. Overall, the environmental improvements seen in Lake Eustis were smaller in magnitude than in Lake Griffin. The relatively moderate improvements in Lake Eustis may be due to the upstream restoration efforts at Lake Apopka, whereas Lake Griffin is likely benefiting from the extensive restoration efforts at EMCA in addition to the upstream efforts (Fulton et al., 2015).

### 2.2 Insect collection

A rapid biodiversity assessment was conducted over five days, August 14th–18th, 2018. These dates were chosen to be in the same subtropical season as Thaxter's visit. Again, although Thaxter's precise methodology is not known, many of the species recorded from Eustis, for example, members of *Autoicomyces*, *Ceratomyces*, *Hydrophilomyces*, *Rhynchophoromyces*, and *Zodiomyces*, are found on nocturnal aquatic beetles, indicating he likely used a light‐based trap as part of his collection methods. Insects were therefore collected using an ultraviolet trapping method. This popular entomological collection method was chosen in order to collect a broad range of taxa (Szentkirályi, 2002; van Wielink & Spijkers, 2013). Given that many insects are nocturnal and given the risk of alligator encounter in and near the perimeter of the lakes, this method was deemed to be both effective and safe. A black light, (2805 DC Light Night Collecting Light, DC, 12 Volt, 15 Watt BL) was set against a white sheet, which was placed approximately 5 m from the water's edge. Insects were collected via aspirator and transferred to 70% ethanol for storage. Three collectors spent three hours at each of the two sites per night, totaling 90 effort hours of collecting. Equal collection effort was made at both sites and the starting location alternated each night between Eustis or EMCA (Appendix 1).

### 2.3 Fungal collection

All collected insects were scanned for infections of Laboulbeniales under a Nikon stereomicroscope at 20–40×. Presence/absence data were recorded for each insect. All insect specimens were identified family level or lower, using Marshall (2006) and are housed at SUNY College of Environmental Science & Forestry (SUNY‐ESF) in Syracuse, New York. Fungal thalli were removed with a micropin and mounted in glycerin using previously described methods (Benjamin, 1971, 1986, 1993). All fungi were identified to species using Thaxter (1896, 1908, 1924, 1926, 1931) and Majewski (1994) and personal correspondence with Weir. Voucher specimens are housed at SUNY‐ESF.

### 2.4 Data and data analyses

Abundance, species richness, and species diversity (Simpson's and Shannon‐Weiner, H’) were calculated for both insects and Laboulbeniales. Comparisons are presented from the two time periods for collections in Eustis (1897 and 2018) as well as between Eustis and EMCA. As this was a case study resulting from a single collection event at each site, actual statistical comparisons could not made as it would have been psuedoreplication.

## 3 RESULTS

**FIGURE 2:**
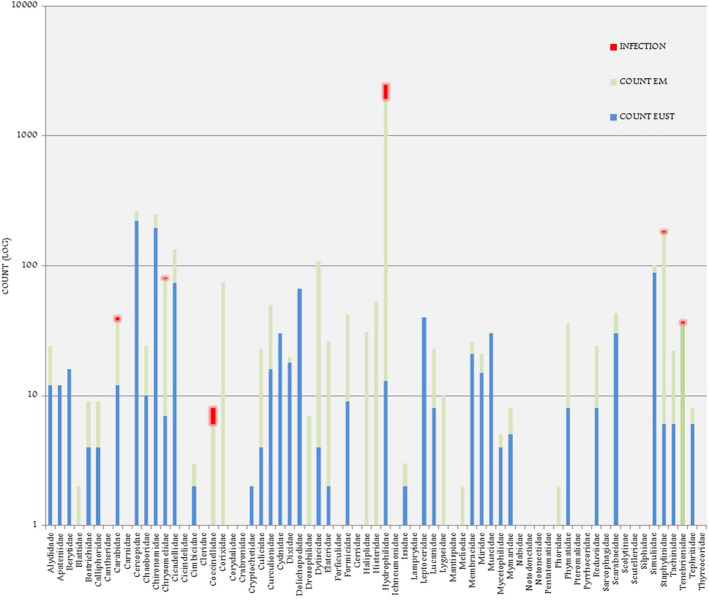
Insect family richness, abundance and infection at EMCA and Lake Eustis. Note that both of the two Coccinellidae individulas collected at Lake Eustis were infected, thus appearing red and not blue

**FIGURE 3:**
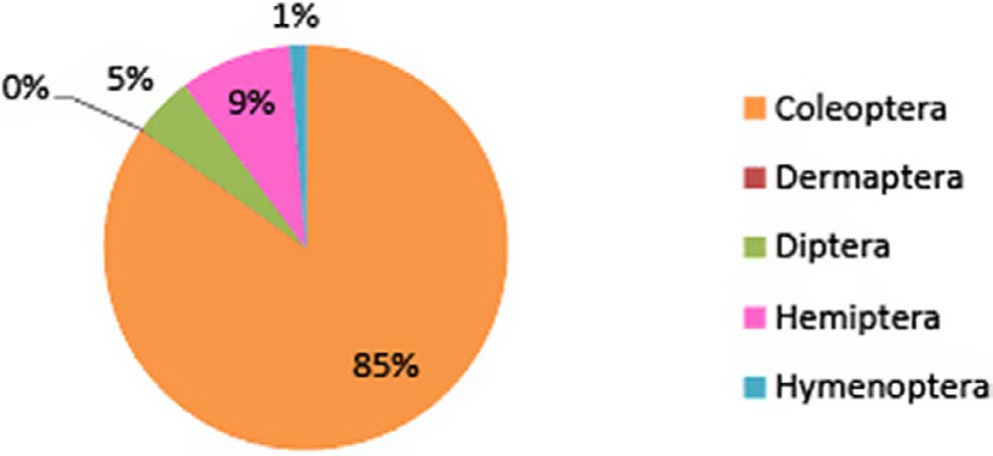
Relative abundance of insects by order from EMCA. The dominant order at EMCA was Coleoptera, hosting all detected species of Laboulbeniales

**FIGURE 4:**
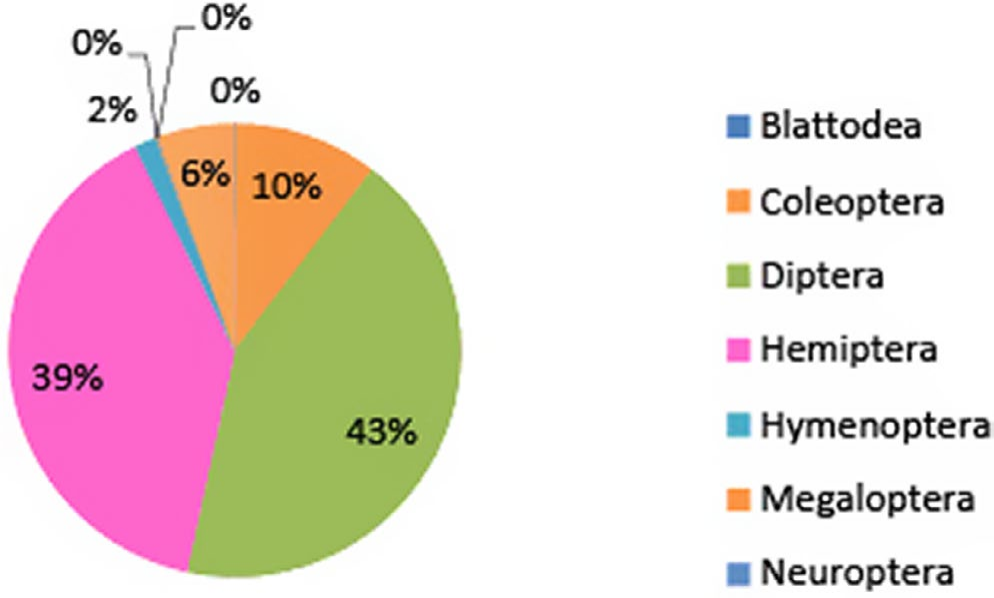
Relative abundance of insects by order from Eustis. Hemiptera and Diptera were the two dominant orders at Eustis

A total of 4022 insects were collected during the rapid assessment (Figure 2). Overall insect abundance was greater at EMCA, with 3001 insects collected, compared to 1021 insects collected from Eustis (Table 1). Insect richness at the family level was comparable between lakes with species from 55 families trapped at EMCA and 56 at Eustis. Species diversity indices were comparable between the two sites, with H’ = 0.44 and D = 0.09 at EMCA and H’ = 0.40 and D = 0.09 at Eustis. The different insect assemblages are presented in Figures 2, 3, 4. The most noteworthy contrast between the lakes was the relative abundance of the family Hydrophilidae (water scavenger beetles), with 1923 individuals collected from EMCA and only 13 from Eustis.

At EMCA, parasite prevalence was 17.6%, with 529 out of 3001 of the insects being host to Laboulbeniales fungi. Comparatively, only ~0.19%, or 2 out of 1021 insects collected from Eustis were host Laboulbeniales. Eleven species of Laboulbeniales were found at EMCA (Table 1): *Ceratomyces filiformis*, *C. longicornis*, *C. mirabilis*, *Chitonomyces* sp., *Hesperomyces virescens*, *Hydrophilomyces gracilis*, *Hy. hamatus*, *Laboulbenia philonthi*, *L*. sp. 1, *L*. sp. *2*, *Zodiomyces vorticellarius*. Only one species, *Hesperomyces virescens*, was found at Eustis. Infections on insects from EMCA all occurred on the following Coleoptera families: Hydrophilidae, Staphylinidae (rove beetles), Coccinellidae (lady beetles), Carabidae (ground beetles), Tenebrionidae (darkling beetles), and Chrysomelidae (leaf beetles). Infections on the two insects from Eustis were both on Coccinellidae. The vast majority of infected insects, 519 out of 529 in total, were members of the Hydrophilidae, with the remaining ten infections occurring on the other families. Relative insect abundance by order at both sites is shown in Figures 3 and 4. The dominant insect orders at Eustis were Diptera and Hemiptera, whereas the dominant insect order at EMCA was Coleoptera, host to all detected Laboulbeniales at the sites.

## 4 OCCURRENCE RECORDS

### 4.1 *Ceratomyces* Thaxt

The genus *Ceratomyces* was established by Thaxter (1892) and currently contains 21 species. Very few publications contain information on *Ceratomyces* since Thaxter's contributions (Bernardi et al., 2014; Goldmann & Weir, 2018; Santamaria, 1999; Shen et al., 2009; Tavares, 1985). Because relatively few contributions have been made to this genus, it is of value to update occurrence records for all species of *Ceratomyces* found during this study. In addition to the data obtained here additional occurrence and range extension data are published for the first time from the collection of Dr. Richard K. Benjamin, which is currently housed in the mycological herbarium at SUNY‐ESF. Further information about these collections, such as host data and precise locality, can be accessed through mycoportal.org.

### 4.2 *Ceratomyces* *ansatus* Thaxt

**FIGURE 5:**
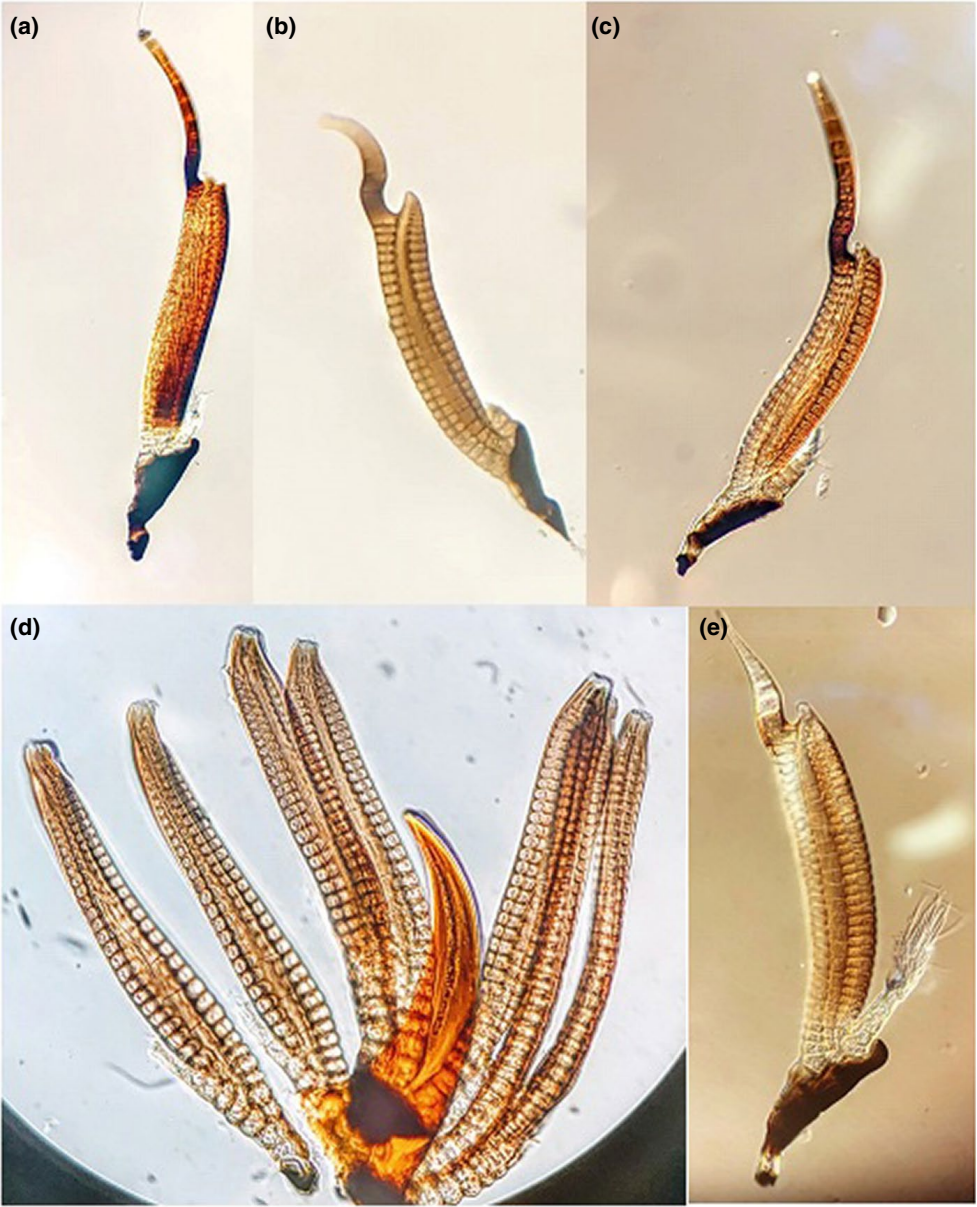
(a) *Ceratomyces longicornis*; (b) *Ceratomyces confusus*; (c) *Ceratomyces mirabilis*; (d) *Ceratomyces filiformis* attached to host claw; (e) *Ceratomyces ansatus*

Specimens examined: USA, FL, Lake County Emeralda Marsh Conservation Area. 28°58′1.46″N 81°48′13.88″W. August 14–18, 2018 on *Tropisternus striolatus*. (Hydrophilidae, Coleoptera), leg. P. Kaishian, [PK1F; PK2F, PK13F]. (Figure 5e).

Remarks: This species has not been formally recorded in North America since its original publication by Thaxter (1908), where he reported the type from Brazil, and a specimen from Eustis, FL. This species was also reported from Argentina (Tavares, 1985; Thaxter, 1931). Weir and Rossi (2001) reported this species from Santa Cruz Dept., Bolivia.

Additional Specimens examined: Within the collection of Dr. Richard Benjamin, currently housed at SYRF, there are heretofore unreported specimens of *Ceratomyces ansatus* collected (in chronological order) from: Jackson, IL, USA, 1909 (RKB 436A, RKB 495A); Morelos, Mexico, 1948 (RKB 1956, RKB 1756A); Alachua, FL, USA, 1954 (RKB 1773A); Musuas, Nicaragua, 1945 (RKB 1987); Turrialba, Costa Rica, 1955 (RKB 1990A); San Antonio, El Salvador, 1957 (RKB 3708E); Porto Alegre, Brazil, Date N/A (RKB 1314).

### 4.3 *Ceratomyces* *confusus* Thaxt

Specimens examined: USA, FL, Lake County Emeralda Marsh Conservation Area. 28°58′1.46″N 81°48′13.88″W. August 14–18, 2018 on *Tropisternus glaber*. (Hydrophilidae, Coleoptera), leg. P. Kaishian, [PK26F; PK27F]. (Figure 5b).

Remarks: This species was first recorded from *Tropisternus glaber* and *T. nimbatus* in Milford, CT, and Kittery Point, ME, USA by Thaxter (1896). It was later reported by Thaxter (1908) in Eustis on several different species of *Tropisternus*.

Additional Specimens Examined: Within the collection of Dr. Richard Benjamin, currently housed at SYRF, there are heretofore unreported specimens of *Ceratomyces confusus* collected (in chronological order) from: Oaxaca and Michoacán, Mexico, 1948 (RKB 1946A, RKB 1948B); San Diego, CA, USA, 1953 (RKB 1598A, RKB 1704D, RKB 1809C, RKB 2247); Dixie, FL, USA, 1954 (RKB 1759); Los Angeles, CA, USA, 1954 (RKB 1774); Chiapas, Mexico, 1964 (RKB 2304B).

### 4.4 *Ceratomyces* *filiformis* Thaxt

Specimens examined: USA, FL, Lake County Emeralda Marsh Conservation Area. 28°58′1.46″N 81°48′13.88″W. August 14–18, 2018 on *Tropisternus glaber*. (Hydrophilidae, Coleoptera), leg. P. Kaishian, [PK4F; PK27F]. (Figure 5d).

Remarks: This widespread species was first recorded from *Tropisternus glaber* and *T. nimbatus* in Milford, CT, Kittery Point, ME, and Arlington, MA, USA by Thaxter (1896). It was later reported by Thaxter (1908, 1931) in Eustis, FL and in TX, USA, as well as Mexico, Guatemala, Argentina, Brazil, and Chile, on several different species of *Tropisternus*, as well as on *Pleurohomos obscurus* from Guatemala.

Additional Specimens Examined: Within the collection of Dr. Richard Benjamin, currently housed at SYRF, there are heretofore unreported specimens of *Ceratomyces filiformis* collected (in chronological order) from: Trinidad and Tobago, 1913 (RKB 1607C00); Orange, FL, USA, 1945 (RKB 1929C); Jalisco, Veracruz, Michoacán, and Morelos, Mexico, 1948 (RKB 1952, RKB 1757, RKB 1953A, RKB 1952, RKB 1950, RKB 1948A, RKB 1946B, RKB 1938B); Champaign, IL, USA, 1950 (RKB 1239); Puno Dept., Peru, 1951 (RKB 1943A); Albany, WY, USA, 1951 (RKB 1795); San Diego, CA, 1953 (RKB 1598B, RKB 1704B); Alachua, FL, USA, 1954 (RKB 1753F); Turrialba, Costa Rica, 1955 (RKB 1990C); San Antonio, El Salvador, 1957 (RKB 3708B); Marzan Dept., Honduras, 1957 (RKB 3511G); Chiapas, Mexico, 1964 (RKB 2304C, RKB 2303B); and Yellowstone National Park, WY, USA, 1971 (RKB 2810D, RKB 2810E, RKB 2810F).

### 4.5 *Ceratomyces* *longicornis* Thaxt

Specimens examined: USA, FL, Lake County Emeralda Marsh Conservation Area. 28°58′1.46″N 81°48′13.88″W. August 14–18, 2018 on *Tropisternus glaber*. (Hydrophilidae, Coleoptera), leg. P. Kaishian, [PK4F]. (Figure 5a).

Remarks: This species was described from Eustis, FL, USA on *Tropisternus* sp. and has not been reported since (1931) in or outside the original locality.

### 4.6 *Ceratomyces* *mirabilis* Thaxt

Specimens examined: USA, FL, Lake County Emeralda Marsh Conservation Area. 28°58′1.46″N 81°48′13.88″W. August 14–18, 2018 on *Tropisternus glaber*. (Hydrophilidae, Coleoptera), leg. P. Kaishian, [PK3F]. (Figure 5e).

Remarks: This species, considered by Thaxter to be the most common member of the genus in the world, was first recorded from *Tropisternus glaber* and *T. nimbatus* in Milford, CT, Arlington, MA, and Kittery Point, ME, USA by Thaxter (1896). Later, Thaxter (1931) lists the following places from which this species has been found: New England and FL, USA, Mexico, Guatemala, Costa Rica, Cuba, Trinidad, French Guiana, Brazil, Amazonas, Argentina, and Chile. All examined species occur on several different species of *Tropisternus*, as well as on *Pleurohomos obscurus* from Guatemala.

Additional Specimens Examined: Within the collection of Dr. Richard Benjamin, currently housed at SYRF, there are heretofore unreported specimens of *Ceratomyces mirabilis* collected (in chronological order) from: IL, USA, 1907, 1908 (RKB 136A, RKB 136B); Lee, TX, 1908 (RKB 132B, RKB 135B); Trinidad and Tobago, 1913 (RKB 1607B); Puno, Peru, 1918 (RKB 1818A, RKB 1818B); Pima and Santa Cruz, AZ, USA, 1935, 1936 (RKB 3508A, RKB 3937B, RKB 3938A, RKB 3938B); Orange, FL, USA, 1945 (RKB 1929B); San Luis Potosí, Morelos, Oaxaca, Nayarit, Veracruz, Mexico, 1948 (RKB 1954B, RKB 1953B, RKB 1948C, RKB 1947C, RKB 1935, RKB 1936B, RKB 1937A, RKB 1938A RKB 1955); Bernalillo, USA, 1949 (RKB 3934B); Champaign, IL, USA, 1950 (RKB 1238C, RKB 490A, RKB 594B, RKB 594C); Angol, La Araucanía and Coquimbo Dept., Chile, 1950 (RKB 9151B, RKB 1951C, RKB 1934, RKB 1933B, RKB 1932); Chiapas, Mexico, 1950 (RKB 1531); San Diego, CA, USA, 1953, 1954, (RKB 1704C, RKB 1809D, RKB 1810); Putnam, FL, USA, 1954 (RKB 3406); Turrialba, Costa Rica, 1955 (RKB 1989B); San Bernardino, CA, USA, 1956 (RKB 2038C); Santa Barbara, CA, USA, 1957 (RKB 2050); Puebla and Michoacán, Mexico, 1957 (RKB 2079, RKB 2078, RKB 3706C, RKB 3709B); Morazan Dept., Honduras, 1957 (RKB 3511B, RKB 3511C, RKB 3511D, RKB 3511E, RKB 3511F); Loja, Ecuador, 1958 (RKB 2064B, RKB 2064A); Barinas, Venezuela, 1958 (RKB 2256A, RKB 2256B, RKB 2256C, RKB 2256D, RKB 2256E); San Diego, CA, USA, 1960 (RKB 3515A, RKB 3515B, RKB 3515C); Chiapas, Mexico, 1964 (RKB 2303, RKB 2304A); and Yellowstone National Park, WY, USA, 1971 (RKB 2810A, RKB 2810B, RKB 2810C); OR, USA, Date N/A (RKB 1958A).

### 4.7 *Hydrophilomyces* Thaxt

The genus *Hydrophilomyces* was erected by Thaxter (1908) and now contains 16 species. Subsequent contributions to this genus have been made by Picard (1910), Spegazzini (1915), Thaxter (1931), Sarna and Milewska (1977), Majewski (1974, 1983, 1994), Huldén (1983), Rossi (1990), Santamaria (2006, 2020), Tavares (1985). Two species of the genus—*H. reflexus* and *H. rhynchophorus*—were originally described from Eustis, FL, by Thaxter and were first placed within the genus *Ceratomyces* (1900) before moved to *Hydrophilomyces* in 1908. These species were not re‐collected in this study, however, two other members of the genus—*H. gracilis* and *H. hamatus—*were collected. As with *Ceratomyces* because relatively few contributions have been made to this genus, it is of value to update occurrence records for all species of *Hydrophilomyces* found during this study. In addition to the data obatined here additional occurrence and range extension data are published for the first time.

### 4.8 *Hydrophilomyces* *hamatus* Majewski

**FIGURE 6:**
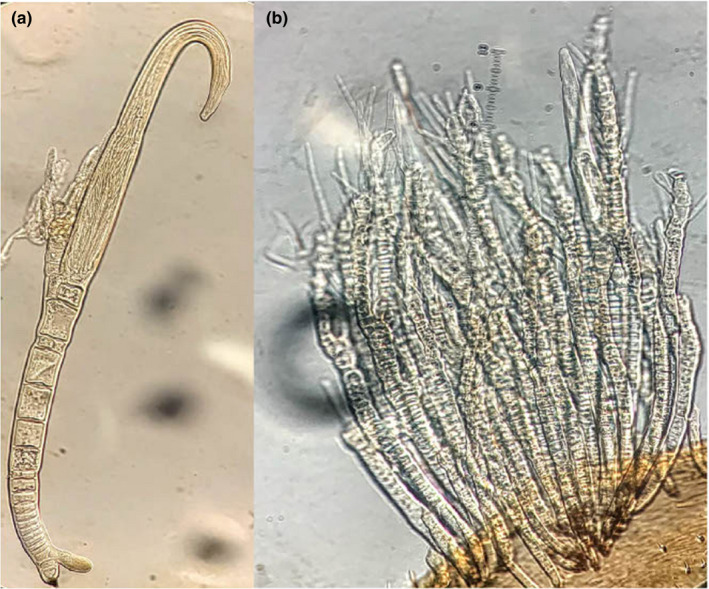
(a) *Hydrophilomyces hamatus*; (b) a cluster of *Hydrophilomyces gracilis*

Specimens examined: USA, FL, Lake County Emeralda Marsh Conservation Area. 28°58′1.46″N 81°48′13.88″W. August 14–18, 2018 on *Cercyon* sp. (Hydrophilidae, Coleoptera), leg. P. Kaishian, [PK11F] (Figure 6a).

Remarks: This species was previously only recorded from Sierra Leone (Rossi, 1990), British Isles (Weir & Beakes, 1993), and Poland (Majewski, 1994), making this is the first formal report of the species occurring in this hemisphere.

Additional Specimens Examined: Within the collection of Dr. Richard Benjamin, currently housed at SYRF, there are heretofore unreported specimens of *H. hamatus* collected (in chronological order) from: Ocala, FL, USA, 1962 (RKB 2974, RKB 2973); Tavares, FL, USA, 1967 (RKB 2968); Thomas, NE, USA, 1967 (RKB 2470, RKB 2469).

### 4.9 *Hydrophilomyces* *gracilis* Majewski

Specimens examined: USA, FL, Lake County Emeralda Marsh Conservation Area. 28°58′1.46″N 81°48′13.88″W. August 14–18, 2018 on *Phaenonotum* sp. (Hydrophilidae, Coleoptera), leg. P. Kaishian, [PK17F; PK23F] (Figure 6b).

**FIGURE 7:**
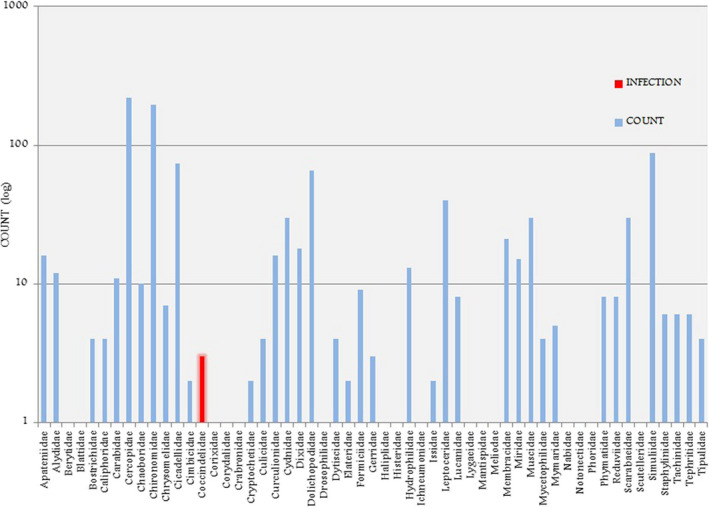
Insect family richness, abundance, and infection at Lake Eustis

**FIGURE 8:**
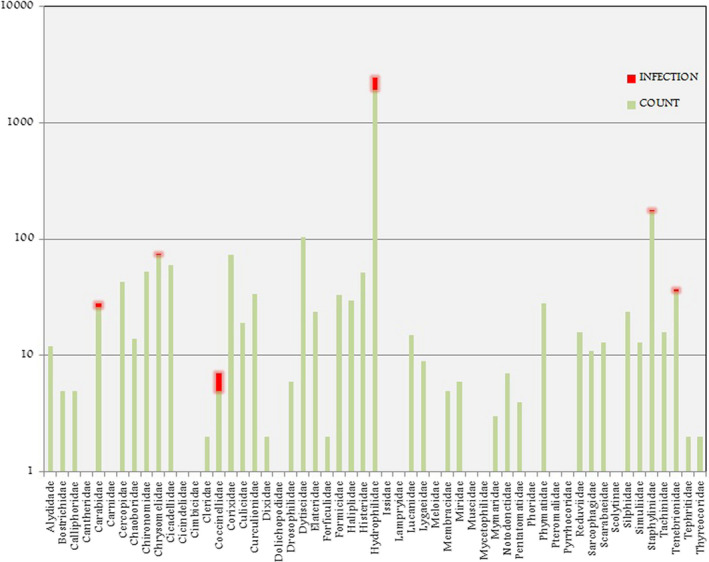
A logarithmic scale graph showing insect family richness abundance and infection from EMCA

Remarks: This species was previously only recorded from Poland (Majewski, 1974, 1994) and Greece (Castaldo et al., 2004), making this the first record in the Western hemisphere (Figures 7 and 8).

## 5 DISCUSSION

Despite being relatively understudied compared with other groups of fungi, the Laboulbeniales possess certain qualities that lend themselves toward being a model group for studies in ecosystem health. For one, these fungi are reasonably visually detectable ectoparasites, which form 3‐dimensional parenchymatous structures, referred to as thalli (Blackwell et al., 2020; Haelewaters et al., 2021; Weir & Hammond, 1997). Additionally, the fungi persist intact on the host for decades if the host is collected and preserved either in by pinning or stored in ethanol. Therefore, entomological collections serve as a tremendous repository for these organisms, allowing researchers to utilize an alternative source for novel taxa, host lists, or habitat associations (Haelewaters et al., 2021; Kaishian & Weir, 2018). Similarly, previous systematic and spatial work by entomologists already exist and can be utilized for the study of this group. Furthermore, as discussed before, some fungi, particularly those that form fleshy sporocarps, may only fruit during extremely narrow and/or sporadic windows of time. Because Laboulbeniales lack a known asexual stage and possess spores that are only briefly viable in the environment (Cottrell & Riddick, 2012; De Kesel, 1995), the presence of their fruiting body in the environment is more reliable and consistent indicator of species presence than many other groups of fungi. The combination of all these factors uniquely positions the Laboulbeniales as a focal group for fungal diversity studies.

Previous studies have begun to explore the potential of Laboulbeniales as indicators. Sugiura et al. (2010) conducted a study of Carabidae beetles and their associated species of *Laboulbenia* across different habitats in central Japan—a riverside, secondary forest and farmland and the microhabitats therein—to quantify insect–fungus interactions at the host assemblage level. This study found that 14/156 or 8.97% of Carabidae collected at the riverside site were infected with *Laboulbenia* thalli; 2/214 or 0.93% in the forest; and 0/161 or 0% at the farmland habitat. This builds evidence that the host habitat partly impacts the prevalence of Laboulbeniales. This study expanded upon studies by Andersen and Skorping (1991) and De Kesel (1996b), which both demonstrated, via field work and experimentation respectively, that host microhabitat impacts occurrence of Laboulbeniales and the presence of host taxa alone does not guarantee presence of fungal counterparts.

In this study, the contrast of the abundance of Hydrophilidae, more specifically the subfamily Hydrophilinae, between sites is of particular note, given that Hydrophilinae are host to most of the fungal infections recorded at EMCA. Because Hydrophilinae are nocturnal and attracted to light (Thorp & Rogers, 2015), it is possible that ambient artificial light from the town of Eustis diminished the relative attraction of the UV light, contributing to the difference in Hydrophilidae abundance between sites. However, given the well‐established and devastating effect of artificial light at night on many nocturnal insects, it is not unreasonable to posit that artificial light introduced with urban development coupled with outright habitat destruction around the perimeter of Lake Eustis and surrounding wetlands disrupted normal biological activities of the Hydrophilinae population, leading to population decline (Owens et al., 2019). The loss of emergent and floating‐leaved vegetation may also play a role in the insect population declines. Such vegetation was relatively abundant at the EMCA collection site as it had been purposefully re‐introduced (Fulton et al., 2015) compared with Eustis where no such efforts have been reported. If the insect population declined, then the associated fungal population may have also declined. At EMCA, the infection rate of the Hydrophilidae population was 20%, but 0 of the 13 Hydrophilinae collected from Eustis were infected. This would perhaps suggest insect population dwindled to levels no longer facilitative of direct transmission of Laboulbeniales, which is thought to be the primary mode of transmission for this group (Cottrell & Riddick, 2012; De Kesel, 1993, 1995; Haelewaters et al., 2015; Nalepa & Weir, 2007; Richards & Smith, 1955; Scheloske, 1969; Weir & Beakes, 1995). In other words, Laboulbeniales require a certain host population density, meaning that Laboulbeniales decline may be a harbinger for insect population decline. The presence or absence of Laboulbeniales may be a proxy for the robustness of the insect population, which in turn may be a proxy for the overall health of the habitat. This raises the question: Is there a quantifiable threshold at which insect population decline leads to extirpation of Laboulbeniales species? Future studies should seek to address this question and build upon and replicate the methodology of this study in numerous habitats in order to garner evidence for the utility of Laboulbeniales as indicators of ecosystem health.

It should be noted that the single species of Laboulbeniales recorded from Eustis, *Hesperomyces virescens*, is a widely dispersed species which occurs on various *Harmonia* species including *Ha. axyridis*, an invasive species in North America. *Ha. axyridis* is now common across the east coast but was not present during Thaxter's original visit (Haelewaters et al., 2015). No other species collected in this study are known to be occurring on invasive insects. For additional discussion of introduced species of Laboulbeniales see Haelewaters et al. (2015).

When considering Laboulbeniales, collection of insects offers a two‐for‐one assessment of insects and fungi, providing a more textured, multikingdom understanding of biodiversity at a given site. In this study, similar richness of insect families was found at both sites, whereas the presence of fungi was markedly different, with a 17.6% infection rate at EMCA and a 0.19% infection rate at Eustis. In this case, without attention paid to the fungal dimension, incorrect conclusions may be drawn about population trends or robustness, and overall biodiversity of these two habitats. A multikingdom assessment consolidates resources and effort, which increases the feasibility of conducting biodiversity monitoring. Making such work more feasible is attractive, given the mounting pressure of climate change and the related impacts on biodiversity. These findings also highlight that specialist organisms, such as the highly host‐specific and obligately associated members of the Laboulbeniales, may be of particular risk for population decline, range restriction, loss of genetic diversity, extirpation, and total extinction in a changing climate (Thomas, 2000; Warren et al., 2001). For such organisms, establishing protected areas and carrying out focused monitoring protocols is of great importance (Chape et al., 2005). (see Table 1A).

## AUTHOR CONTRIBUTION

**Patricia J. Kaishian:** Conceptualization (lead); Data curation (lead); Formal analysis (lead); Funding acquisition (lead); Investigation (lead); Methodology (lead); Project administration (lead); Resources (lead); Software (lead); Supervision (lead); Validation (lead); Visualization (lead); Writing‐original draft (lead); Writing‐review & editing (lead).

### OPEN RESEARCH BADGES

This article has earned an Open Data badge for making publicly available the digitally‐shareable data necessary to reproduce the reported results. The data is available at https://doi.org/10.5061/dryad.47d7wm3f5.

## Data Availability

All insect and fungal material is available for loan upon request from SUNY‐ESF. All insect biodiversity data is archived with Dryad: https://doi.org/10.5061/dryad.47d7wm3f5.

## APPENDIX 1

**TABLE 1A:** Insect family count and infection data from Eustis and EMCA

EUSTIS				EMCA			
Order	Family	Count	Infection	Order	Family	Count	Infection
Blattodea	Blattidae	1	0	Coleoptera	Bostrichidae	5	0
Coleoptera	Bostrichidae	4	0	Coleoptera	Cantheridae	1	0
Coleoptera	Carabidae	11	0	Coleoptera	Carabidae	26	2
Coleoptera	Chrysomelidae	7	0	Coleoptera	Chrysomelidae	73	1
Coleoptera	Coccinellidae	2	2	Coleoptera	Cicindelidae	1	0
Coleoptera	Curculionidae	16	0	Coleoptera	Cleridae	2	0
Coleoptera	Dytiscidae	4	0	Coleoptera	Coccinellidae	5	2
Coleoptera	Elateridae	2	0	Coleoptera	Curculionidae	34	0
Coleoptera	Haliplidae	1	0	Coleoptera	Dytiscidae	105	0
Coleoptera	Histeridae	1	0	Coleoptera	Elateridae	24	0
Coleoptera	Hydrophilidae	13	0	Coleoptera	Haliplidae	30	0
Coleoptera	Lucanidae	8	0	Coleoptera	Histeridae	52	0
Coleoptera	Meliodae	1	0	Coleoptera	Hydrophilidae	1923	519
Coleoptera	Scarabaeidae	30	0	Coleoptera	Lampryidae	1	0
Coleoptera	Staphylinidae	6	0	Coleoptera	Lucanidae	15	0
Diptera	Caliphoridae	4	0	Coleoptera	Meloidae	1	0
Diptera	Chaoboridae	10	0	Coleoptera	Scarabaeidae	13	0
Diptera	Chironomidae	195	0	Coleoptera	Scolytinae	1	0
Diptera	Cryptochetidae	2	0	Coleoptera	Silphidae	24	0
Diptera	Culicidae	4	0	Coleoptera	Staphylinidae	175	3
Diptera	Dixidae	18	0	Coleoptera	Tenebrionidae	36	1
Diptera	Dolichopodidae	66	0	Dermaptera	Forficulidae	2	0
Diptera	Drosophilidae	1	0	Diptera	Calliphoridae	5	0
Diptera	Muscidae	30	0	Diptera	Carnidae	1	0
Diptera	Mycetophilidae	4	0	Diptera	Chaoboridae	14	0
Diptera	Phoridae	1	0	Diptera	Chironomidae	53	0
Diptera	Simuliidae	88	0	Diptera	Culicidae	19	0
Diptera	Tachinidae	6	0	Diptera	Dixidae	2	0
Diptera	Tephritidae	6	0	Diptera	Dolichopodidae	1	0
Diptera	Tipulidae	4	0	Diptera	Drosophilidae	6	0
Hemiptera	Alydidae	12	0	Diptera	Muscidae	1	0
Hemiptera	Berytidae	1	0	Diptera	Mycetophilidae	1	0
Hemiptera	Cercopidae	220	0	Diptera	Phoridae	1	0
Hemiptera	Cicadellidae	74	0	Diptera	Sarcophagidae	11	0
Hemiptera	Cimbicidae	2	0	Diptera	Simuliidae	13	0
Hemiptera	Corixidae	1	0	Diptera	Tachinidae	16	0
Hemiptera	Cydnidae	30	0	Diptera	Tephritidae	2	0
Hemiptera	Gerridae	3	0	Hemiptera	Alydidade	12	0
Hemiptera	Issidae	2	0	Hemiptera	Cercopidae	43	0
Hemiptera	Lygaeidae	1	0	Hemiptera	Cicadellidae	60	0
Hemiptera	Membracidae	21	0	Hemiptera	Corixidae	74	0
Hemiptera	Miridae	15	0	Hemiptera	Issidae	1	0
Hemiptera	Nabidae	1	0	Hemiptera	Lygaeidae	9	0
Hemiptera	Notonectidae	1	0	Hemiptera	Membracidae	5	0
Hemiptera	Phymatidae	8	0	Hemiptera	Miridae	6	0
Hemiptera	Reduviidae	8	0	Hemiptera	Notodonctidae	7	0
Hemiptera	Scutelleridae	1	0	Hemiptera	Pentatomatidae	4	0
Hymenoptera	Crabronidae	1	0	Hemiptera	Phymatidae	28	0
Hymenoptera	Formiciidae	9	0	Hemiptera	Pyrrhocoridae	1	0
Hymenoptera	Ichneumonidae	1	0	Hemiptera	Reduviidae	16	0
Hymenoptera	Mymaridae	5	0	Hemiptera	Thyreocoridae	2	0
Megaloptera	Corydalidae	1	0	Hymenoptera	Cimbicidae	1	0
Neuroptera	Mantispidae	1	0	Hymenoptera	Formicidae	33	0
Trichoptera	Apateniidae	16	0	Hymenoptera	Mymaridae	3	0
Trichoptera	Leptoceridae	40	0	Hymenoptera	Pteromalidae	1	0
